# Integrated database-based Screening Cohort for Asian Nomadic descendants in China (Scan-China): Insights on prospective ethnicity-focused cancer screening

**DOI:** 10.4178/epih.e2023048

**Published:** 2023-04-18

**Authors:** Yuelin Yu, Liying Qiao, Jing Han, Weiwei Wang, Weiwei Kang, Yunjing Zhang, Shu Shang, Ruogu Meng, Lin Zhuo, Siyan Zhan, Yunfeng Xi, Shengfeng Wang

**Affiliations:** 1 Department of Epidemiology and Biostatistics, School of Public Health, Peking University, Beijing, China; 2 Center for Disease Control and Prevention in Inner Mongolia, Hohhot, China; 3 National Institute of Health Data Science, Peking University, Beijing, China; 4 Research Center of Clinical Epidemiology, Peking University Third Hospital, Beijing, China

**Keywords:** Community-based screening cohort, Cancer health disparity, Mongolian, Integrated healthcare databases, Mass screening, Early detection of cancer

## Abstract

Established in 2017, the Screening Cohort for Asian Nomadic descendants in China (Scan-China) has benefited over 180,000 members of a multi-ethnic population, particularly individuals of Mongolian descent compared with the general population (Han ethnicity), in the Inner Mongolia Autonomous Region, China. This cohort study aims to evaluate the effectiveness of cancer screening and serve as a real-world data platform for cancer studies. The 6 most prevalent cancers in China are considered—namely, breast, lung, colorectal, gastric, liver and esophageal cancer. After baseline cancer risk assessments and screening tests, both active and passive follow-up (based on the healthcare insurance database, cancer registry, the front page of hospital medical records, and death certificates) will be conducted to trace participants’ onset and progression of cancers and other prevalent chronic diseases. Scan-China has preliminarily found a disproportionately lower screening participation rate and higher incidence/mortality rates of esophageal and breast cancer among the Mongolian population than among their Han counterparts. Further research will explore the cancer burden, natural history, treatment patterns, and risk factors of the target cancers.

## INTRODUCTION

Most screening programs are one-size-fits-all, despite their high efficiency in identifying high-risk and early-onset populations and cost-effectiveness in reducing cancer incidence and mortality [1-3]. Racial/ethnic minorities worldwide, compared to the general populations, are reported to have lower and less timely uptake and completion of screening [4-10], along with a higher risk of cancer health disparities due to genetic, geographic, and socioeconomic factors [11-13]. In Western countries, Asian minorities have among the severest patterns of these alarming disparities [6]. However, this issue has been persistently overlooked on the multicultural Asian continent.

Mongolians, who descend from Asian nomads inhabiting grassland areas, are among the largest ethnic minorities in East Asia (over 10 million population worldwide, including 6.3 million Chinese residents). Mongolians’ traditional dietary habits are characterized by highly caloric, salty food and high consumption of red meat and dairy products to accommodate their migratory patterns [14]. Although they currently live mixed with the general population (Han ethnicity) in China, Mongolians’ distinctive living patterns, together with genetic factors conferring susceptibility [15,16] and cultural beliefs [17] inherited from their ancestors, may contribute to pessimistic uptake and poor effectiveness of screening. However, Mongolian-oriented screening programs have not been initiated, and studies have not focused on cancer disparities between Mongolians and the general population in China. These gaps have led to cancer health disparities being persistently unaddressed.

The Screening Cohort for Asian Nomadic descents in China Inner Mongolia Autonomous Region (Scan-China) project is the first and largest screening program tailored for the Mongolian ethnicity, initiated in the Inner Mongolia Autonomous Region of China. Inner Mongolia is the area with the largest population of the Mongolian ethnicity globally, wherein Mongolians constitute the second-largest ethnicity (19.3% of the total population) [18]. Derived from the Cancer Screening Program in Urban China (detailed information can be seen in previous relative literature [19,20] and in the Supplementary Material 1), Scan-China offers cancer risk assessments and screening tests for the 6 most prevalent cancers in urban areas (lung, breast, liver, esophageal, gastric and colorectal). Scan-China aims to evaluate the effectiveness of screening, particularly for ethnic minorities, to describe the natural history and explore risk factors of the targeted cancer types and prevalent comorbidities, and to portray treatment patterns among different ethnicities, all contributing to addressing cancer health disparities across ethnicities.

**Figure 1.**
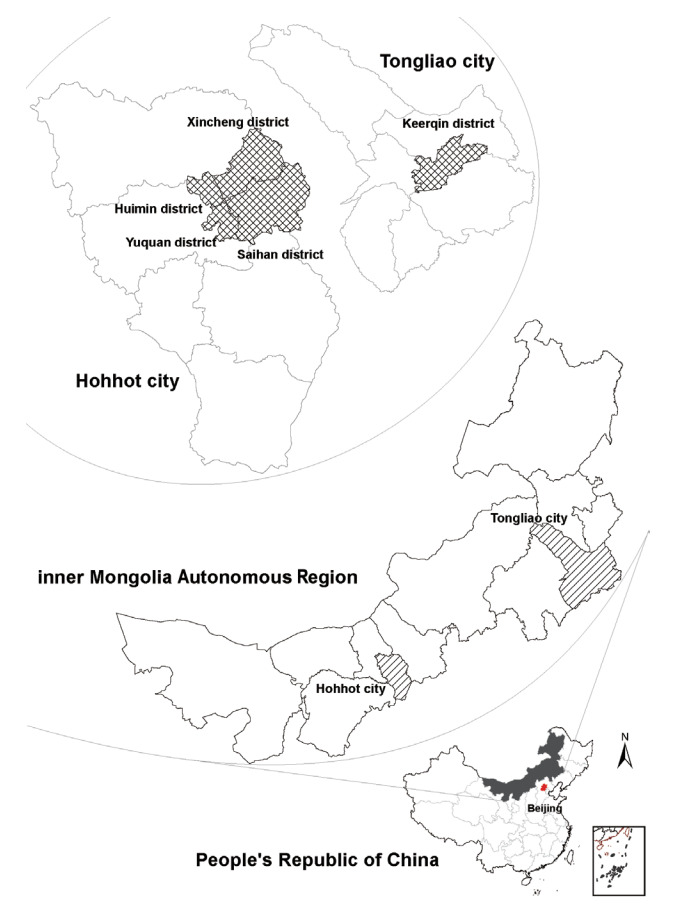
Spatial distribution of participants in the baseline survey of Screening Cohort for Asian Nomadic descents in China Inner Mongolia Autonomous Region.

Scan-China targets urban residents in 5 districts of 2 major cities (Xincheng District, Huimin District, Yuquan District, and Saihan District in the city of Hohhot; Keerqin District in the city of Tongliao) with the largest population (over 1 million per city) in Inner Mongolia (Figure 1). The districts were selected based on population size, representativeness of multiple ethnicities (Han, Mongolian, and other ethnic minorities living mixed together), and the feasibility of project implementation considering healthcare resources and hospital collaborations. In detail, Hohhot is the provincial capital and Tongliao is one of the birthplaces of the Mongolian ethnicity, respectively representing developed (top-level gross domestic product) and developing (medium-level gross domestic product) levels of healthcare resources in Inner Mongolia [18].

## STUDY PARTICIPANTS

**Figure 2.**
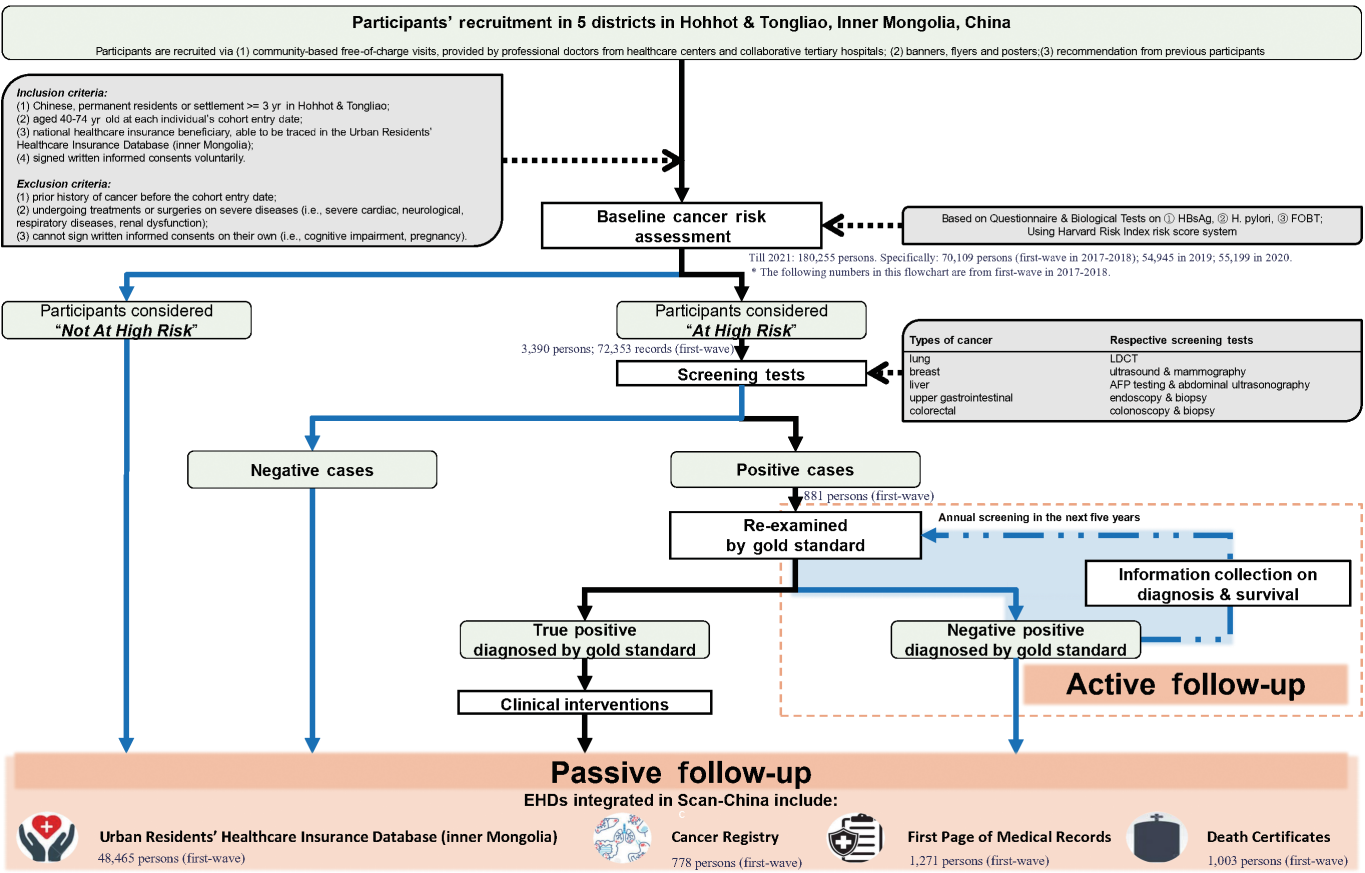
Flowchart of Screening Cohort for Asian Nomadic descents in China Inner Mongolia Autonomous Region (Scan-China). HBsAg, hepatitis B surface antigen; H. pylori, Helicobacter pylori; FOBT, Fecal Occult Blood test; LDCT, low-dose computed tomography; AFP, alpha-Fetoprotein; EHDs, electronic healthcare databases. The numbers attached to each section of the flowchart are from first-wave in 2017-2018, able to show one complete annual procedure of Scan-China. Data in later waves is undergoing processed and not presented here.

This dynamic cohort was planned to benefit 30,000 new participants annually. Participants who meet the following inclusion criteria would be recruited in Scan-China after a baseline cancer risk assessment: (1) Chinese residents of the catchment areas, or with a minimum residence of 3 years in Hohhot or Tongliao, (2) aged 40-74 years old at the cohort entry date, (3) being a community-dwelling national healthcare insurance beneficiary, (4) having voluntarily signed a written informed consent form to participate in Scan-China. Individuals of all ethnicities have equal rights and chances to participate in Scan-China. Eligible individuals are voluntarily enrolled and not restricted by ethnicity. More detailed inclusion and exclusion criteria are shown in the Supplementary Material 1 and Figure 2.

Follow-up is conducted both actively and passively. All participants in Scan-China will be passively followed via annual linkage of the baseline results of risk assessment and screening tests to multi-source electronic health data (EHD) databases in Inner Mongolia. The EHD databases of Scan-China include the Urban Residents’ Healthcare Insurance Database (URHID), the Cancer Registry (CaR), the front page of hospital medical records (FPMR), and death certificates (DCs), all officially governed by the Inner Mongolia Center for Disease Control and Prevention.

Data linkage and integration are conducted using personal identity numbers, which are de-identified during data analysis for privacy protection. Standardized coding systems are used across the EHD databases of Scan-China. The diagnoses of diseases and causes of death are coded using the International Classification of Diseases, 10th revision (ICD-10). The prescriptions in URHID are coded using the Anatomical Therapeutic Chemical (ATC) system. Moreover, specific validation of the accuracy of EHD-sourced diagnoses has been conducted independently by 2 clinical experts, who checked the diagnoses of a certain portion of random samples from the whole cohort population (an initial assessment found about 94% accuracy in the diagnoses of cardiovascular diseases).

The active follow-up is tailored for participants who receive positive screening results for any cancer via phone calls or in-house visits, as well as medical records for subsequent confirmation of their status of cancer onset. Clinical experts from collaborative tertiary hospitals will conduct gold-standard examinations to diagnose cancer. Participants who receive positive screening results but negative examination findings will undergo annual re-screening for the next 5 years. Participants with confirmed diagnoses of cancer will be recommended to visit doctors for professional treatment.

### Ethics statement

The project was approved by the Institutional Review Board (IRB) of Ethics Committee of Inner Mongolia Autonomous Region Center for Disease Control and Prevention (IRB No. NMCDCIRB2021001). Informed consent was confirmed by the IRB. The project has been submitted for registration in the China Clinical Trial Registration Center. The recruited participants all signed written informed consent at baseline. The authors affirm that the human research participants provided informed consent for the publication of all results involved in this paper.

## MEASUREMENTS

Information throughout the screening is collected via a baseline questionnaire and biological tests for cancer risk assessment, screening tests and blood samples if necessary, and active and passive follow-up via EHD databases. A detailed statistical analysis plan for the analyses that will be conducted and what will be reported when the follow-up period is extended to 10 years or 20 years can be seen in the Supplementary Material 1. Cancer-related information is mainly collected during follow-up, involving diagnoses, treatments, and details on cancer onset and survival status (Table 1).

### Baseline cancer risk assessment: questionnaire survey for all participants

All eligible participants are required to engage in cancer risk assessments via a paper-based questionnaire with instructions from trained staff (Table 1). The assessment is based on the Harvard Risk Index [21-23]. For each participant, the information collected at baseline includes socio-demographic characteristics, behaviors and environmental/occupational exposures to cancer-related risk factors, psychological conditions, and personal and family history of diseases. Details on lifestyle habits such as food preferences regarding temperature and flavor are also collected, filling an insufficiency in most previous screening cohorts.

### Baseline screening and blood sample collection: biochemical tests for the high-risk population

Only high-risk participants according to the baseline assessment are recommended to receive screening tests for the respective targeted cancers. All screening tests are provided by collaborating hospitals, free of charge, and are conducted by physicians with over 5 years of clinical experience. Meanwhile, an expert panel from the National Cancer Center of China has been assembled as the third party to provide consultation if physicians are unsure of reaching a positive or negative classification. For participants at high-risk for liver cancer, upper gastrointestinal cancer, and colorectal cancer, a 5-mL blood sample per person is collected prior to the above screening tests. Each participant’s screening test results and details on pathology reports are archived both physically and electronically in the screening database [22,24].

### Electronic health data-integrated follow-up strategy: annual dynamic updates for all participants

A detailed description of the core variables available in Scan-China databases is shown in Table 1 and Supplementary Material 2. In brief, information on disease diagnoses, prescriptions, hospitalizations, and medical expenses throughout an individual’s entire course of hospital visits will be available in the URHID. The FPMR offers details related to clinical diagnoses and in-hospital treatments, while the CaR concentrates on tumor-related information, including cancer onset, clinical status, and pathological findings. The survival status, causes of death, and date of death will be monitored using DCs. The passive follow-up will be annually conducted to dynamically update each participant’s health condition.

## KEY FINDINGS

Scan-China was established on January 1, 2017 and residents could enter the cohort dynamically. By December 31, 2021, Scan-China has benefited 180,255 people (about 11% Mongolian) in Inner Mongolia (70,109 at the first-wave baseline completed by December 31, 2018), with an average increase of 4,500 new participants annually. For 48,471, 792, and 1,004 participants, disease onset and ongoing disease status were reported in the URHID (over 2.3 million records on diagnoses of the Scan-China participants were captured from January 1, 2017 to December 31, 2021), CaR, and DC, respectively.

After baseline questionnaire validation, successful linkage across EHDs, and data cleaning, 68,349 Han and Mongolian participants at the first-wave baseline were included in the analysis, with median (interquartile, IQR) age of 55.12 years (IQR, 47.82 to 62.93; 52.7% female, 11.7% Mongolian). Compared with the Han population, the Mongolian population showed significantly larger proportions of consumption of red meat (38.5 vs. 30.8%); hot (26.6 vs. 20.9%), salty (32.1 vs. 23.3%) and high-oil (30.7 vs. 23.4%) foods; and alcohol (28.5 vs. 21.5%) (p< 0.001 for all) (Table 2). The Mongolian population had a higher prevalence of chronic diseases (30.0 vs. 23.8% for chronic respiratory diseases, 26.10 vs. 19.03% for upper gastrointestinal diseases, 17.3 vs. 12.9% for lower gastrointestinal diseases, 32.3 vs. 25.0% for hepatobiliary diseases; p< 0.05 for all) and higher risky in all types of cancer (over 20 vs. less than 20%, p< 0.001 for all).

Differences in cancer incidence according to sex and ethnicity were specifically described (Table 3). The highest overall 3-year cumulative incidence was of breast cancer (4.28 per 1,000 persons; 95% confidence interval [CI], 3.61 to 4.94), followed by lung cancer (3.47; 95% CI, 3.03 to 3.91), colorectal cancer (2.87; 95% CI, 2.47 to 3.27), gastric cancer (1.52; 95% CI, 1.23 to 1.81), liver cancer (1.14; 95% CI, 0.89 to 1.39) and esophageal cancer (0.66; 95% CI, 0.47 to 0.85). Compared with the Han population, Mongolians showed a higher incidence of esophageal cancer (2.26; 95% CI, 0.59 to 3.93 vs. 1.16; 95% CI, 0.76 to 1.55 in male; 0.21; 95% CI, 0.00 to 0.61 vs. 0.13; 95% CI, 0.00 to 0.25 in female) and breast cancer (4.73; 95% CI, 2.80 to 6.65 vs. 4.21; 95% CI, 3.50 to 4.92). The top 3 cancers in terms of mortality were lung cancer (1.23; 95% CI, 0.97 to 1.49), liver cancer (0.82; 95% CI, 0.60 to 1.03) and gastric cancer (0.45; 95% CI, 0.29 to 0.61). Among all targeted cancer types, male Mongolians showed higher mortality from esophageal cancer (1.29; 95% CI, 0.03 to 2.55 vs. 0.53; 95% CI, 0.26 to 0.79).

## STRENGTHS AND WEAKNESSES

Scan-China is the first EHD-integrated dynamic screening cohort targeting multiple ethnicities in Inner Mongolia. With the aim of addressing poor screening effectiveness among ethnic minorities, Scan-China is a unique platform for popularizing applicable health interventions for the Mongolian minority.

Cancer cohorts integrated with EHD databases show greater cost-effectiveness and time-effectiveness [25-27]. The current findings from Scan-China demonstrated accurate linkage across the baseline population and respective EHD databases. In particular, the capture rate of passive follow-up through the claims database reached 92.2%, which is better than the extant high-quality passive EHD follow-up [28], indicating the feasibility and high efficiency of the EHD-integrated follow-up strategy. Although data quality remains a stubborn challenge for almost all real-world studies [29,30], the multi-source EHD databases enable Scan-China to achieve information validation, timestamp selection, and progression tracking of diseases. More importantly, Scan-China sets a framework for the linkage and integration of heterogeneous EHD databases in the scope of a cancer screening cohort, advancing beyond the CHERRY study [31], which utilized inherently linked EHD on cardiovascular diseases.

Another distinctive merit of Scan-China is that it presents information on comorbidities, complications, and treatment patterns among Mongolian patients. It should be mentioned that traditional Mongolian medicine accounts for a majority of treatment strategies among this population, in parallel with Western medicine and traditional Chinese medicine. Scan-China will shed light on cancer-related treatment priorities regarding drug effectiveness and safety. For example, cardiovascular complications have been increasingly reported as a major drug adverse reaction during chemotherapy [32]. Differences in treatment patterns among the Mongolian ethnicity, in comparison with the general population, might provide insights into how to ameliorate disparities in the prognosis of cancer and other prevalent cardiovascular diseases.

Nonetheless, the project has some limitations. First, Scan-China only targets urban residents and lacks representativeness of the rural population in consideration of study feasibility. Furthermore, the baseline questionnaire was only answered by participants who volunteered to take part in the study. This might have generated selection bias. Second, the baseline information on cancer-related risk factors (such as lifestyle habits) was all self-reported, which induced unavoidable recall bias. Third, the inclusion criteria in terms of the age range might have caused information loss on early-life exposures. Third, overdiagnosis, overtreatment, and misinterpretation of clinical data may have taken place [33]. However, all the aforementioned limitations are inherent to most screening cohorts’ design and cannot be avoided [34]. Moreover, problems with EHD quality are inherently unavoidable. Previous studies have reported that cancer incidence has been underestimated [35,36]. However, combining multi-source EHD databases might complement the completeness and reliability of records. Furthermore, the 3-year cumulative cancer incidence from Scan-China showed smaller differences between males and females than reported in the previous literature [37,38]. This may have been partly because the denominator used for incidence calculation was only composed of the first-wave population at the current preliminary stage. Alternatively, the larger differences could be explained by the inclusion of all cancer patients in other studies, rather than high-risk groups in the age range of 40-74 years or participants in cancer screening programs. Therefore, the findings from Scan-China need to be cautiously generalized in the context of comparable screening programs, population proportions, and data sources.

## DATA ACCESSIBILITY

Scan-China is not an open-access database. The data utilized in its future studies will be available in de-identified form upon reasonable request, with approval from the expert panel of Scan-China, the Inner Mongolia Autonomous Region Center for Disease Control and Prevention, and the Ethics Committee of National Cancer Center/Cancer Hospital, China Academy of Medical Sciences and Peking Union Medical College. Collaborations and external investigations of the Scan-China dataset are welcomed to make further contributions to cancer health promotion. The expert panel of Scan-China will contact you via e-mail if your application is considered meaningful (with application materials including the study protocol, statistical analysis plan, and contribution statement) and data use is approved by the above committees.

## Figures and Tables

**Table 1.** Summary of core information collected in Screening Cohort for Asian Nomadic descendants

Category		Measures	Core variables	
			Examples	No. of variables
Information collected at baseline				111
	Socio-demographics		Birthdate; sex; ethni group; birth place; education; marital status	14
	Behaviors and exposures	Dietary habits	Average consumption of fresh vegetables, fruits, red meat, dairy products, grains, etc. in the past 2 years; food preference on the temperature, flavor, etc.	9
		Lifestyle	Nicotine & alcohol consumption (status, consuming intensity [frequency & amount], timespan for quitting); tea consumption & physical activity (status, consumption intensity)	10
		Occupational exposure	Occupational exposures to hazardous substances; type of dangerous substances	3
		Environmental exposure	Outdoor air pollution; indoor fumes exposure to heating/cooking/passive smoking (type & exposure intensity)	5
	Psychological condition		Exposure to psychological trauma (serious illness or death of family members/friends, family breakdown, etc.); mental depression	2
	Health condition	Body shape	Height; weight; waist circumference	3
		History of biological tests	Pathogenic tests for hepatitis B surface antigen, Helicobacter pylori, fecal occult blood test	4
		History of diseases	Diagnosis of malignant tumors; chronic respiratory diseases (tuberculosis, chronic bronchitis, asthma, emphysema, etc.); gastrointestinal diseases on esophagus, stomach and intestines (gastroesophageal reflux, superficial gastritis, ulcers, polyposis, ulcerative colitis and Crohn's disease, etc.); hepatobiliary diseases (chronic hepatitis B/C, liver cirrhosis, fatty liver, gallstones, etc.); cardiovascular and metabolic diseases (hyperlipidemia, hypertension, diabetes, etc.)	37
		Family history of malignant tumors	Tumor onset sites (lung, breast, liver, esophagus, stomach, colorectum, female breast/ovary, etc.); type of kinship with tumor-incident relatives (parents, grandparents, siblings, cousins, etc.); age of tumor onset; number of tumor-incident relatives	7
		Reproductive and gynecological history (female only)	Age at menarche/menopause/first childbirth; regularity of menstrual cycles; history of child delivery (abortion, stillbirth, living); history of breastfeeding; history of benign breast diseases; history of treatments (medications, surgery, etc.) on female genitalia	10
Information collected during active follow-up				16
	General information		Status of follow-up (drop-out, withdrawal, loss)	1
	Cancer onset	Clinical diagnosis	Primary site of tumor; type of pathology; clinical & pathological TNM classification	5
	Treatment		Treatment status (ongoing, not treated, unknown)	2
	Survival		Status of last contact (alive or not); date of last contact	2
			Date of death; cause of death	2
Information collected from passive follow-up				
	Survival		Status; date of death; underlying & direct causes of death	5
	Diagnoses	Clinical	Date of clinical diagnosis; name of diseases; clinical TNM classification for cancer diagnoses	18
		Pathological	Date of pathological diagnosis; name of diseases; pathological TNM classification for cancer diagnoses; pathological anatomical information (lesion sites, pathological stage, number of tumors, onset sequence of primary tumors, age at tumor onset, etc.)	10
	Treatments	Type of treatments	Medication (chemotherapy, TCM, etc.); invasive therapies (surgery, biopsy, etc.); non-invasive procedures (ultrasonography, X-ray, etc.); radiotherapy, etc.	1
		Medications	Trade/general name; route of administrations; date of prescription; dosing intensity (frequency & amount & timespan)	5
		Operations & procedures	Date of operation; name of operation; operational sites	6
	Costs and reimbursements	Healthcare services	Type of medical insurance, type of visits (general outpatient, referral, emergency, pharmacy, etc.), hospital information (general/TCM-specified type, primary/secondary/tertiary level, etc.)	17
		Costs	Total medical expenses, costs on medications/procedures/surgeries/laboratory tests, etc.	10
	Hospitalization	Admission	Date of admission/referral, clinical department at admission/referral	6
		Discharge	Date of discharge, primary/secondary diagnosis at discharge (disease name & International Classification of Diseases-10 code)	15

TNM, tumor node metastasis; TCM, traditional Chinese medicine.

**Table 2.** Baseline characteristics of the first-wave Han and Mongolian populations in Screening Cohort for Asian Nomadic descendants (n=68,349)^1^

Characteristics			Han ethnicity	Mongolian ethnicity	p-value
No. of participants at first-wave baseline			60,380 (88.3)	7,969 (11.7)	
	Age at first wave, mean±SD (yr)		55.77±9.07	54.19±8.53	<0.001
	Sex				<0.001
		Male	28,529 (47.2)	3,103 (38.9)	
		Female	31,851 (52.7)	4,866 (61.1)	
	Education				<0.001
		Junior high school or lower	32,839 (54.4)	3,368 (42.3)	
		Senior high school or higher	27,541 (45.6)	4,601 (57.7)	
	Martial status				<0.001
		Currently single	1,653 (2.7)	282 (3.5)	
		Currently married	58,727 (97.3)	7,687 (96.5)	
	Equipped with household heating				<0.001
		Yes	58,720 (97.2)	7,874 (98.8)	
		No	1,660 (2.7)	95 (1.2)	
	Type of household heating fuels				<0.001
		Cleaner fuels	55,158 (93.9)	7,083 (89.9)	
		Coal	3,532 (6.0)	784 (10.0)	
		Others	30 (0.0)	7 (0.1)	
	Type of household cooking fuels				<0.001
		Cleaner fuels	58,482 (96.9)	7,632 (95.8)	
		Coal	1,734 (2.9)	310 (3.9)	
		Others	164 (0.3)	27 (0.3)	
Dietary habits^2,3^					
	Average consumption (/wk) of fresh vegetables				<0.001
		Never	3,179 (5.3)	268 (3.4)	
		Not much	41,600 (68.9)	5,348 (67.1)	
		Meet the recommended amount	15,601 (25.8)	2,353 (29.5)	
	Average consumption (/wk) of fresh fruits				<0.001
		Never	3,873 (6.4)	444 (5.6)	
		Not much	42,466 (70.3)	5,385 (67.6)	
		Meet the recommended amount	14,041 (23.2)	2,140 (26.8)	
	Average consumption (/wk) of red meat				<0.001
		Never	2,521 (4.2)	191 (2.4)	
		Not much	39,260 (65.0)	4,709 (59.1)	
		Meet the recommended amount	18,599 (30.8)	3,069 (38.5)	
	Average consumption (/wk) of coarse grains				<0.001
		Never	5,108 (8.5)	817 (10.2)	
		Not much	41,799 (69.2)	5,288 (66.4)	
		Meet the recommended amount	13,473 (22.3)	1,864 (23.4)	
	Preference for food temperature				<0.001
		Hot	12,594 (20.9)	2,123 (26.6)	
		Moderate	46,105 (76.4)	5,624 (70.6)	
		Cold	1,681 (2.8)	222 (2.8)	
	Preference for food flavor				<0.001
		Salty	14,053 (23.3)	2,561 (32.1)	
		Moderate	41,927 (69.4)	4,686 (58.8)	
		Plain (light seasoning)	4,400 (7.3)	722 (9.1)	
	Preference for fat and oil content				<0.001
		High	14,147 (23.4)	2,450 (30.7)	
		Moderate	42,282 (70.0)	4,836 (60.7)	
		Little	3,951 (6.5)	683 (8.6)	
	Smoking status				<0.001
		Never	46,071 (76.3)	5,696 (71.5)	
		Ex-smoker	1,554 (2.6)	272 (3.4)	
		Current smoker	12,755 (21.1)	2,001 (25.1)	
	Alcohol consumption				<0.001
		Never	46,324 (76.7)	5,481 (68.8)	
		Ex-drinker	1,087 (1.8)	220 (2.8)	
		Current drinker	12,969 (21.5)	2,268 (28.5)	
	Tea consumption				<0.001
		Never	40,071 (66.4)	4,472 (56.1)	
		Not often	1,525 (2.5)	278 (3.5)	
		Regular	18,784 (31.1)	3,219 (40.4)	
	Having regular physical exercises		22,605 (37.4)	3,070 (38.5)	0.061
	Body mass index, mean±SD (kg/m^2^)		24.02±2.79	24.54±3.13	<0.001
Self-reported prevalence of chronic diseases					
	Chronic respiratory diseases		14,345 (23.8)	2,389 (30.0)	<0.001
		Tuberculosis	1,781 (12.4)	319 (13.4)	0.205
		Chronic bronchitis	12,734 (88.8)	2,136 (89.4)	0.380
		Emphysema	3,549 (24.7)	243 (10.2)	<0.001
		Asthma	5,503 (38.4)	769 (32.2)	<0.001
	Upper gastrointestinal diseases		11,491 (19.0)	2,080 (26.1)	<0.001
		Gastroesophageal reflux	2,972 (25.9)	586 (28.2)	0.028
		Superficial gastritis	9,365 (81.5)	1,746 (84.0)	0.007
		Atrophic gastritis	2,795 (24.3)	380 (18.3)	<0.001
		Gastric ulcers	4,629 (40.3)	831 (40.0)	0.808
		Duodenal ulcers	2,322 (20.2)	370 (17.8)	0.011
		Gastric polyposis	1,916 (16.7)	258 (12.4)	<0.001
	Lower gastrointestinal diseases		7,787 (12.9)	1,379 (17.3)	<0.001
		Intestinal polyposis	2,796 (37.6)	392 (30.6)	<0.001
		Ulcerative colitis and Crohn's disease	6,470 (87.0)	1,152 (89.9)	0.004
	Hepatobiliary diseases		14,980 (25.0)	2,537 (32.3)	<0.001
		Fatty liver	13,589 (89.6)	2,316 (88.3)	0.046
		Gallstone	6,857 (45.2)	1,264 (48.1)	0.006
		Chronic hepatitis B	1,086 (7.2)	211 (8.1)	0.095
		Chronic hepatitis C	788 (5.2)	130 (5.0)	0.702
		Liver cirrhosis	3,085 (20.4)	224 (8.6)	<0.001
	Cardiovascular and metabolic diseases				
		Hypertension	7,991 (67.9)	1,722 (69.4)	0.142
		Diabetes mellitus	2,465 (20.9)	511 (20.6)	0.724
		Hyperlipidemia	6,484 (55.1)	1,601 (64.5)	<0.001
	Family history of diseases		10,476 (17.3)	2,211 (27.7)	<0.001
		Lung cancer	5,334 (48.7)	1,123 (48.4)	0.766
		Esophageal cancer	1,608 (14.7)	399 (17.2)	0.003
		Gastric cancer	2,681 (24.5)	534 (23.0)	0.129
		Liver cancer	4,718 (43.2)	874 (37.7)	<0.001
		Colorectal cancer	2,164 (19.8)	325 (14.0)	<0.001
		Breast cancer	4,013 (12.6)	898 (18.4)	<0.001
	Assessed at high risk for^4^:				
		Lung cancer	13,249 (21.9)	2,216 (27.8)	< 0.001
		Esophageal cancer	9,796 (16.2)	1,828 (22.9)	< 0.001
		Gastric cancer	11,151 (18.5)	2,055 (25.8)	< 0.001
		Liver cancer	9,237 (15.3)	1,634 (20.5)	< 0.001
		Colorectal cancer	11,426 (18.9)	2,008 (25.2)	< 0.001
		Breast cancer	6,458 (20.3)	1,295 (26.6)	< 0.001

Values are presented as number (%). SD, standard deviation; RR, relative risk.

1 Two participants’ ethnicities were missing and 1,760 participants’ ethnicities were neither Han nor Mongolian, and were thus excluded from the analysis; Only Han and Mongolian participants were included in the above analysis (n=68,349).

2 For the categories of “average consumption (per week)” in different types of food: “not much” of fresh vegetables, fresh fruits, red meat, coarse grains was respectively defined as <2,500 g, <1,250 g, <350 g, and <500 g/wk.

3 Meeting the recommended amount of fresh vegetables, fresh fruits, red meat, coarse grains as respectively defined as ≥2,500 g, ≥1,250 g, ≥350 g, and ≥500 g/wk.

4 Assessed at high risk in the targeted 6 types of cancer was defined as a RR ≥1.50 using an established risk score system based on the Harvard Risk Index.

**Table 3.** Incidence and mortality density^1^ of each targeted cancer type for the first-wave Han and Mongolian populations in Screening Cohort for Asian Nomadic descendants (n=68,349)^2^

Variables		New patients, n (%)		Cumulative incidence (95% CI), per 1,000 people			
		Han	Mongolian	Male		Female	
				Han	Mongolian	Han	Mongolian
Cumulative incidence from 2019 to 2021							
	Any type of cancer	1,887 (88.7)	241 (11.3)	32.14 (30.10, 34.19)	31.90 (25.72, 38.09)	30.45 (28.57, 32.34)	29.18 (24.45, 33.91)
	Lung cancer	215 (90.7)	22 (9.3)	4.63 (3.84, 5.41)	3.22 (1.23, 5.22)	2.61 (2.05, 3.17)	2.47 (1.07, 3.86)
	Esophageal cancer	37 (82.2)	8 (17.8)	1.16 (0.76, 1.55)	2.26 (0.59, 3.93)	0.13 (0.00, 0.25)	0.21 (0.00, 0.61)
	Gastric cancer	95 (91.3)	9 (8.6)	2.42 (1.85, 2.99)	2.58 (0.79, 4.36)	0.82 (0.50, 1.13)	0.21 (0.00, 0.61)
	Liver cancer	69 (88.5)	9 (11.5)	1.61 (1.15, 2.08)	2.58 (0.79, 4.36)	0.72 (0.43, 1.02)	0.21 (0.00, 0.61)
	Colorectal cancer	174 (88.8)	22 (11.2)	4.24 (3.49, 5.00)	4.19 (1.92, 6.46)	1.66 (1.22, 2.12)	1.85 (0.64, 3.06)
	Breast cancer	134 (85.3)	23 (14.6)	-	-	4.21 (3.50, 4.92)	4.73 (2.80, 6.65)
Cumulative mortality from 2017 to 2021							
	Any type of cancer	299 (92.0)	26 (8.0)	6.73 (5.78,7.68)	6.12 (3.38, 8.87)	3.36 (3.36, 3.36)	1.44 (0.37, 2.50)

CI, confidence interval.

1 None of the differences in incidence/mortality density between Han and Mongolian ethnicity, regardless of the sex category showed statistical significance (all p-values >0.05).

2 Two participants’ ethnicities were missing and 1,760 participants’ ethnicities were neither Han nor Mongolian, and were thus excluded from the analysis; Only Han and Mongolian participants were included in the above analysis (n=68,349).
