# Supplementary material for: Integrated database-based Screening Cohort for Asian Nomadic descendants in China (Scan-China): Insights on prospective ethnicity-focused cancer screening
Source: Epidemiol Health. 2023 Apr 18;45:e2023048. doi: 10.4178/epih.e2023048 (PMC10593583; doi:10.4178/epih.e2023048)
Supplement: Supplement Material 1. [file epih-45-e2023048-Supplementary-1.docx]

**Supplementary information of Screening Cohort for Asian Nomadic descents**

**in China INner mongolia Autonomous region**

**(Scan-China Project)**

**Catalogue**

[Administrational framework of Scan-China 1-1](#_Toc126780866)

[Main purpose of Scan-China: 1-3](#_Toc126780867)

[Statistical analysis plan 1-3](#_Toc126780868)

[Data Accessibility 1-5](#_Toc126780869)

[How to get the official information of Scan-China: 1-5](#_Toc126780870)

[How to apply for the data of Scan-China for scientific explorations: 1-5](#_Toc126780871)

[Data Application form 2-1](#_Toc126780872)

[1. Author registration 2-2](#_Toc126780875)

[2. Proposed title 2-2](#_Toc126780876)

[3. Contact person 2-2](#_Toc126780877)

[4. Proposal for data application 2-2](#_Toc126780878)

[5. Study context 2-3](#_Toc126780879)

[6. Declarations of interest 2-3](#_Toc126780880)

[7. Applicants' responsibilities 2-3](#_Toc126780881)

[8. Contact applicants 2-4](#_Toc126780882)

# Administrational framework of Scan-China

Scan-China is a screening program under the framework of Cancer Screening Program in Urban China (CanSPUC).

*CanSPUC is the nationwide cancer screening system in China,* *initiated from 2012 by National Cancer Center/Cancer Hospital, China Academy of Medical Sciences and Peking Union Medical College, in support of China National Health Commission and the Ministry of Finance. So far, CanSPUC has covered 26 provinces in China to serve urban residents who live in the participating regions to get access to the targeted six cancer screening (breast cancer, lung cancer, colorectal cancer, gastric cancer, liver cancer and esophageal cancer). Participants aged 40 to 74 will firstly be approached by trained staff to undergo cancer risk assessment. This preliminary step before cancer screening also acts as one health promotion campaign to raise the public awareness of cancer screening on the community level. Only participants being assessed as high risk will undergo the according respective cancer screening tests in the consideration of limited healthcare resources and program feasibility. This screening protocol of CanSPUC is in consistency in all covered cities.*

For Scan-China, it is tailored for the Mongolian ethnicity in the Inner Mongolia Autonomous Region. Under the framework of CanSPUC, which has been elaborated in previous literature (i.e., Guo, L., et al. 2019. *Cancer medicine*, *8*(16), 7098–7107; Guo, L. W., et al. 2020. *JAMA network open*, *3*(11), e2019039). Participants are recruited annually through three main ways: (1) community-based free-of-charge visits provided by professional doctors from healthcare centers and collaborative tertiary hospitals, (2) banners, flyers and posters sent out in public areas, (3) recommendation from participants.

**The inclusion criteria of Scan-China participants:**

Individuals who meet the following eligibility criteria would be recruited in Scan-China: (1) must be Chinese residing in the catchment areas, or with minimum residence of 3 years in Hohhot city or Tongliao city, (2) aged 40-74 years old at their individual cohort entry date, (3) being community-dwelling national healthcare insurance beneficiary, (4) signed the written informed consents on Scan-China voluntarily.

**The exclusion criteria of Scan-China participants:**

Individuals with any conditions in the following would be excluded: (1) prior history of cancer before the cohort entry date, (2) undergoing treatments or surgeries on severe diseases, for example, severe cardiac, neurological, respiratory diseases, renal dysfunction, etc. (3) unwilling or unable to consent/ respond to the baseline questionnaire (i.e., with cognitive impairment, pregnancy). The Scan-China screening is accessible to all eligible national healthcare insurance beneficiaries regardless of language, ethnicity, literacy, or any other social bias.

**Statements on Participants Equivalence:**

The selected cities of Scan-China to carry out the screening programs were Hohhot and Tongliao. Both cities and their districts in the two cities were selected based on population size, representativeness of multi-ethnicities (Han, Mongolian and other ethnic minorities living mixed together) and feasibility of project’s implementation considering healthcare resources and hospital collaborations.

All population in the selected regions who meet both the inclusion and exclusion criteria will be included in Scan-China, voluntarily. To be specific, eligible participants need to have national healthcare insurance. This national healthcare insurance is the national-governed social insurance system in China, established to compensate residents and workers for the economic losses caused by the risk of diseases. The national healthcare insurance is available to all Chinese, regardless of their ethnicities. Since Scan-China focuses on urban regions, eligible population is defined as Chinese residing or working in the catchment urbans. To be mentioned, according to the official data from Inner Mongolia Healthcare Security Bureau, the coverage of national healthcare insurance in Inner Mongolia has reached 95% (Work summary of Inner Mongolia Healthcare Security Bureau in 2021. https://www.nmg.gov.cn/zwgk/zdxxgk/ghjh/jzqk/202203/t20220309_2014781.html); (Statistical Bulletin of 2021 National Economic and Social Development of Inner Mongolia Autonomous Region. <https://www.nmg.gov.cn/tjsj/sjfb/tjsj/tjgb/202202/t20220228_2010485.html>). Therefore, national healthcare insurance has benefited almost all Chinese residents in Inner Mongolia. This means the inclusion criterion on ‘being national healthcare insurance beneficiary’ will not generate substantial bias on ethnic groups. The reason why we stressed the eligible participants to be national healthcare insurance beneficiary is to passively follow up the recruited participants using the urban residents healthcare insurance database and other electronic healthcare databases. The person ID registered to have national healthcare insurance is unique and acts as the linking key across different electronic health databases. This passive follow-up strategy will efficiently track participants’ health conditions and identify positive cases after the baseline cancer risk assessment and screening tests. Therefore, we guarantee that this criterion will not generate bias and will enable participants to benefit from individual-level long-term health services, such as completion of the screening tests and follow-up using electronic health databases.

# Main purpose of Scan-China:

- To describe incidence and mortality, drug utilization, comorbidities and treatment patterns of prevalent cancer and other important chronic diseases for the Mongolian ethnicity, compared with the majority Han population;
- To explore risk factors of these diseases via developing and validating individual prediction models;
- To evaluate cost-effectiveness of this EHD-based screening strategy using health economic models.

# Statistical analysis plan

**Descriptive analysis**

The primary analysis is to describe incidence and mortality, drug utilization and comorbidities of prevalent cancer and other important chronic diseases for the Mongolian ethnicity, compared with the majority Han population. Based on the primary outcomes, the secondary analysis is to explore and summarize risk factors of these diseases via developing and validating individual prediction models. Furthermore, cost-effectiveness of this EHD-based screening strategy will be evaluated using health economic models.

After data cleaning, descriptive analysis on the summary of study population will be first conducted, including sample size and sociodemographic characteristics at the annual baseline risk assessment, overall and group-specific high-risk rates, incidence and mortality rates on respective type of cancer, participation and adherence rates at the screening and active follow-up section, cohort attrition rates, as well as matching and under-report rates across targeted EHD databases. Incidence and mortality rates of different diseases will be calculated using the ratio of number of new disease-onsetting patients (or deaths caused by diseases of interest) to the corresponding person-years. Person-years is calculated based on timespan from the date of entry to (1) the diagnosis date of diseases of interest, (2) date of censor or (3) death, or (4) the end date of this cohort (outcome date is defined as the earliest occurrence date recorded in the Scan-China databases among the former four outcomes). For participants in each specific wave, incidence and mortality rates can also be calculated using accumulated incidence in the specific time periods.

For example,

$$Incidence of all sites=\frac{No. of new cancer patients of all sites}{\begin{aligned} person years from the date of cohort entry \\ to the outcome date \end{aligned}}$$

The representativeness of cohort participants will be examined by comparison on characteristic core variables between the successfully linked population in any EHD databases (i.e., the claims database) versus unlinked population.

Point estimates and the corresponding 95% confidence interval (CI) will be reported for all descriptive results. Number and percentage will be used for categorical variables, while mean (median) and standard deviation (interquartile range, IQR) will be used for normal (or skew) distributed continuous variables. Statistical difference between groups will be tested using chi-square test or Fisher's exact test for categorical variables, while independent sample t-test (or Kruskal-Wallis rank-sum test) for normal (or skew) distributed continuous variables.

**Association analysis between health outcomes and risk factors**

For the secondary analysis, multiple logistic regression will be conducted to explore risk factors for dichotomous outcomes (i.e., whether diseases of interest onset or not). Cox proportional hazards regression will be conducted for time-to-event outcomes (i.e., all-cause and cancer-specific mortality) to test cancer health disparities between the Mongolian ethnicity and the general population. Regression models will be adjusted using propensity score methods for controlling confounders such as sociodemographic factors (i.e., sex, age, education), history of diseases (i.e., whether having hypertension, diabetes), psychological (i.e., whether having diagnosis on mental health disorders), physical (i.e., average time spent on sports per week) and behavioral conditions (i.e., smoking, drinking), etc. Odds ratios (OR) and relative risks (RR) will be respectively reported for logistic regression and cox regression, along with the corresponding 95% CI.

**Development of clinical prediction models**

Based on extant cancer prediction models for general population, Mongolian-specific prediction models on each targeted type of cancer will be developed with the inclusion of new predictors selected from Scan-China. Predictive capability of the new models on respective cancer types will be compared using discrimination and reclassification and clinical performance will be evaluated using C statistic, calibration, reclassification indexes (i.e., net reclassification improvement, integrated discrimination improvement) and statistical plots.

**Sensitivity analysis**

Sensitivity analysis will be performed among different groups (i.e., ethnicity, age, sex) to assure the validity and stability of results throughout all statistical analysis if necessary.

**Analytical software:**

Data analysis can be run by expert panel of Scan-China and approved applicants by using any statistical software (i.e., R software, STATA, Python), which are licensed and free of conflicts of interest.

# Data Accessibility

## How to get the official information of Scan-China:

Applicants can view the cohort information of Scan-China from the website of China Cohort Consortium (<http://chinacohort.bjmu.edu.cn/project/149/?action=detail>). China Cohort Consortium the biggest and most official cohort information sharing forum in China.

From the website, applicants will get the overall introduction of the Scan-China project, available of the registry information (i.e., project leader, contactor, funding, date of establishment), study population (i.e., sampling, accumulated number of participants, inclusion criteria, exclusion criteria), extant scientific explorations, strengths and limitations, etc.

All information posted on this website is open to all scientists worldwide.

## How to apply for the data of Scan-China for scientific explorations:

Collaborations and external investigations on Scan-China dataset are welcomed to make more contributions to cancer health promotions. Scan-China is not an open-access database. Data utilized in its future studies will be available in the form of de-identification on reasonable request, in the premise of approvals from the expert panel of Scan-China, Inner Mongolia autonomous region Center for disease control and prevention, and the Ethics Committee of National Cancer Center/Cancer Hospital, China Academy of Medical Sciences and Peking Union Medical College.

**After completing the application form for Scan-China data utilization, please email the completed form to Yunfeng Xi, Principal Director, Scan-China Project:** [**xiyunfeng210@163.com**](mailto:xiyunfeng210@163.com)**.**

The expert panel of Scan-China will contact you via e-mail once your application is considered meaningful (with an application list of study protocol, statistical analysis plan, contribution statement, etc.) and the data is approved by the above committees.

**
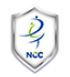
Screening Cohort for Asian Nomadic descents in China INner mongolia Autonomous region**

**(Scan-China Project)**

# Data Application form

Version 1.0, January 2023

Please complete all sections in full of this form, to outline your proposal for data application of Scan-China.

Email the completed form to Yunfeng Xi, Principal Director, Scan-China Project: [xiyunfeng210@163.com](mailto:xiyunfeng210@163.com).

**Data Protection**

The personal data included in this form will be used to complete your applicant profiles if the application is accepted.

Both successful and unsuccessful submissions may be archived for the Scan-China data applicant’s records.

Please note that your names and academic/professional affiliations will be circulated to the expert panel of Scan-China, considering this data application proposal.

🞏 By submitting this form, we give Scan-China permission to process the data included here.

## IMPORTANT: Disclosure of Conflicts of interest

Confirm whether any member of the applicant team has a potential conflict of interest.

If your data application is accepted, Scan-China will request a full Declaration of Interest from each member of the author team.

| Essential checks before submission of data application:  - We have searched for ongoing, completed researches and published literature that has been allowed to use the data of Scan-China, and we can confirm that this proposal has not been covered by another existing research having been registered by Scan-China. - We have checked that this proposal falls within the scope of Scan-China. - We understand that all authors must follow the data protection criteria of Scan-China. - We have informed the expert panel of Scan-China of any potential conflict of interest. |
| --- |

| Author registration | |
| --- | --- |
| To enable Scan-China staff to identify and contact with you, please list the email addresses of your primary applicants. | |
| Applicant 1 | Email address |
| Applicant 2 | Email address |
| Add other rows as required for other applicant team members. | |

| Proposed title Your proposal should not overlap with an existing ongoing, completed researches or published literature that has been allowed to use the data of Scan-China.  You must clearly state the key problem you want to resolve using Scan-China data in your titles, including but not limited to the following key elements:   - [Population] e.g. the Mongolian women - [Intervention/Exposure] e.g. participate in cancer screening - [Compare group] e.g. the Mongolian versus the Han (general) population - [Outcomes] e.g. die of lung cancer | |
| --- | --- |
| Title: |  |

| Contact person Author who will take responsibility for the data application and its according research, and communicate with Scan-China throughout data utilization; does not need to be the first listed author. | |  |
| --- | --- | --- |
| Name: |  | |

| Proposal for data application | |
| --- | --- |
| Why is it important to do this study using Scan-China data? | Why are you proposing to apply for Scan-China data? For example, is it particularly topical to utilize Scan-China data in your study at the present time?  Please use the ‘Study context’ section below to state if this study would form part of a Masters or Doctorate, or of a larger research project. |
| Description of the condition: |  |
| Description of the key variables you want to utilize of Scan-China: | Give a short statement on main databases and according variables in each database of Scan-China you want to apply. e.g. Baseline cancer risk assessment database (Variable A1, Variable B1, Variable C1); Baseline lung screening database (Variable A2, Variable B2, Variable C2). |
| How the variables might work: | Give a short statement of variables of interest you will be used as intervention, or outcomes in your study, e.g. to use Variable A (nominal), Variable B(ordinal), Variable C(ratio)… in the death registry database as our outcomes. |
| Study objectives: | Give a short statement of the primary aim of your study, e.g. to explore the relationship between certain exposure and outcome of your interest. |
| Types of study: | Outline the types of study you will conduct. |
| Participants: | Outline the inclusion and exclusion criteria of participants from Scan-China to be studied. Consider demographic factors, the stage of follow-up (e.g. index date, waves), the type/stage of disease/condition. |
| Intervention/Exposure: | Outline the details of the intervention/exposure you wish to investigate. Are there variations (e.g. intensity, combinations) you wish to exclude? |
| Comparison: | What will the intervention be compared to, e.g. what population, exposure level, no intervention. |
| Primary outcomes: | List the primary outcomes you will measure. Specify how your primary outcomes may be measured, e.g. the type of scale or count likely to be used, the accessible variables can be sourced from Scan-China database, and the follow-up wave of the measurement. |
| Secondary outcomes: | List the secondary outcomes you will measure. Specify how your secondary outcomes may be measured, e.g. the type of scale or count likely to be used, the accessible variables can be sourced from Scan-China database, and the follow-up wave of the measurement. |
| Subgroup analyses: | Outline any subgroups you plan to investigate, e.g. subgroups of the population. |
| Other information: | Outline any other factors you plan to consider in your protocol, or other information you would like to provide, e.g. relevance to consumers, how this study complements other published literature using Scan-China.  (If there are no related completed or ongoing studies, please explain why it is important to do this study.) |
| Related literature: |  |

| Study context | |
| --- | --- |
| Is the study subject to any specific funding? |  |
| Would the study form part of your postgraduate study, or of a larger research project? |  |

| Declarations of interest |
| --- |
| Before your data application can be authorized, each author in your study team must declare any relevant financial interest from the three years prior to the date of this submission. There must be a majority of non-conflicted team members for any particular application and the lead (first) author must have no conflicts. |
| **Do any members of the author team authors have a potential conflict of interest?** Yes 🞏 No 🞏 |
| If yes, you should discuss these potential conflicts with the Scan-China’s Principal Director before submitting this form.  Failure to disclose potential conflicts at this stage, or at any point during your studies using Scan-China data, may lead to it being conflicted to rejection for publication at a later date as your study being contrary to the Scan-China data protection criteria. |

| Applicants' responsibilities |
| --- |
| By completing this form, you accept responsibility for applying, using the approved Scan-China data, and publishing related studies’ outcomes generating from Scan-China data, in accordance with Scan-China’s data protection criteria. The expert panel of Scan-China will provide support to assist with the data utilization.  Scan-China has the right to reject data utilization at any stage throughout your application as well as your later data utilization, if your study intrude the Scan-China data protection criteria.  You accept responsibility for maintaining and sharing the study findings from Scan-China data, in light of new evidence, comments and criticisms, and other developments based on need. |
| I understand and agree to the commitment required to apply and use the Scan-China data.  **Signed on behalf of the study members:** |
| **Form completed by:** |
| **Date:** |

| Contact applicants Applicants who will take responsibility for the protocol and later studies, and communicate with the Scan-China expert panel throughout data application and utilization; no need to be the first listed author – please adjust numbering above. | |  |
| --- | --- | --- |
| Applicant 1 | | |
| Full name and qualifications: *e.g.* *Dr Xue Wang, PhD* |  | |
| Job title: *e.g. Registrar* |  | |
| Affiliation: *e.g. Third Hospital, Peking University* |  | |
| Roles and responsibilities: e.g. draft the protocol, data processing, variables selection, run the analysis, draft study report |  | |
| Applicant 2 | | |
| Full name and qualifications: e.g. Dr Xue Wang, PhD |  | |
| Job title: e.g. Registrar |  | |
| Affiliation: e.g. Third Hospital, Peking University |  | |
| Roles and responsibilities: e.g. draft the protocol, data processing, variables selection, run the analysis, draft study report |  | |
